# Constructing Statistical Intervals for Small Area Estimates Based on Generalized Linear Mixed Model in Health Surveys

**DOI:** 10.4236/ojs.2022.121005

**Published:** 2022

**Authors:** Yan Wang, Xingyou Zhang, Hua Lu, Janet B. Croft, Kurt J. Greenlund

**Affiliations:** 1 Division of Population Health, National Center for Chronic Disease Prevention and Health Promotion, Centers for Disease Control and Prevention, Atlanta, GA, USA; 2 Office of Compensation and Working Conditions, U.S. Bureau of Labor Statistics, Washington, DC, USA

**Keywords:** Bayesian Estimation, Behavioral Risk Factor Surveillance System, Bootstrapping, Monte Carlo Simulation, Small Area Estimation

## Abstract

Generalized Linear Mixed Model (GLMM) has been widely used in small area estimation for health indicators. Bayesian estimation is usually used to construct statistical intervals, however, its computational intensity is a big challenge for large complex surveys. Frequentist approaches, such as bootstrapping, and Monte Carlo (MC) simulation, are also applied but not evaluated in terms of the interval magnitude, width, and the computational time consumed. The 2013 Florida Behavioral Risk Factor Surveillance System data was used as a case study. County-level estimated prevalence of three health-related outcomes was obtained through a GLMM; and their 95% confidence intervals (CIs) were generated from bootstrapping and MC simulation. The intervals were compared to 95% credential intervals through a hierarchial Bayesian model. The results showed that 95% CIs for county-level estimates of each outcome by using MC simulation were similar to the 95% credible intervals generated by Bayesian estimation and were the most computationally efficient. It could be a viable option for constructing statistical intervals for small area estimation in public health practice.

## 1. Introduction

A variety of model-based small area estimation (SAE) methods have been developed and applied to health survey data to generate estimates of health-related outcomes for small geographic areas in recent years [1] [2]. Among these methods, the generalized linear mixed model (GLMM, also called multilevel or hierarchical model) has gained in popularity [3] [4] [5] [6] [7] because it combines different sources of information and error. The parameters from GLMM can be applied to the target small area’s demographic subgroups *via* post-stratification to generate a point estimate for the outcome of interest. To take into account the uncertainty arising from the models, a statistical interval around the estimate is usually constructed.

A credible interval can be drawn directly by Hierarchical Bayesian estimation. In a hierarchical Bayesian model, the unknown parameter is treated as a random variable by giving it a probability distribution and a prior distribution. The posterior distribution of the parameter could be simulated through Markov Chain Monte Carlo (MCMC) samples and consequently a posterior distribution of small area estimate is produced. However, this approach is computationally intensive for large datasets with complex data structures, such as the nationwide Behavioral Risk Factor Surveillance System (BRFSS). In the frequentist paradigm, bootstrapping is a common approach for statistical inference purposes when the true distribution of the statistic of interest is unknown. It has been used to approximate the distribution of the small area estimates [8] [9] [10] and has been shown to make an improvement on the coverage accuracy of confidence intervals [11]. Monte Carlo simulation is another useful tool to generate sample statistics by using point estimates of model parameters and their asymptotic covariance matrix of these estimates [12]. However, these two approaches’ performance has not been evaluated in the context of statistical interval construction of prediction through real complex health surveys.

BRFSS is a common survey used in small area estimation for indicators of chronic diseases, health-related behaviors, and health preventive services. An appropriate approach for constructing statistical intervals using BRFSS can help health agencies or local health departments optimatize their capacity and understand how reliable the estimate is. This study is designed to compare 95% statistical intervals for small area estimates by different approaches using Florida 2013 BRFSS data, which had large sample sizes in all 67 counties. GLMMs combining unit- and area-level covariates were constructed to generate both state- and county-level estimates *via* post-stratification for three selected health-related outcomes; simulated their distributions by using bootstrapping and MC simulation approaches, respectively; and were compared with those based on hierarchical Bayesian estimation via MCMC. The article continues in Section 2 with a presentation of MRP framework and details in 95% interval construction by different approaches. Results are presented and discussed in Sections 3 and 4, respectively. Conclusions are drawn in Section 5.

## 2. Materials and Methods

### 2.1. Data Source

The 2013 Florida BRFSS was a cross-sectional survey data and a part of the nationwide BRFSS (https://www.cdc.gov/brfss/annual_data/annual_2013.html). The county ID was obtained through a Data Use Agreement with the U.S.’s Centers for Disease Control and Prevention, Division of Population Health, Population Health Surveillance Branch. Because it was designed to better estimate county-level prevalence of personal health behaviors that contribute to morbidity and mortality among adults (≥18 years) in Florida by increasing the sample size in each of the 67 counties, it could provide county-level direct estimates for the selected indictors, which was of research interest to the present study. Over 34,000 interviews were completed statewide in the 2013 calendar year, with a target sample size of 500 completed surveys in each county. In the present study, we selected two chronic diseases and one health related behavior measures, self-reported doctor-diagnosed chronic obstructive pulmonary disease (COPD, 7.6%), self-reported binge drinking (17.6%), and self-reported doctor-diagnosed arthritis (26.0%), because they were common indictors but with different prevalence levels at the Florida state level, and also have great public health intervention importance. Their direct survey estimates at the state or county levels for 2013 were obtained from the Florida BRFSS website (https://www.floridahealth.gov/statistics-and-data/survey-data/behavioral-risk-factor-surveillance-system/_documents/2013county/index.html). The data were weighted to the respondent’s probability of selection by county, as well as age, sex, marital status, race/ethnicity, education level, and housing type. Details of 2013 Florida BRFSS methods and the health-related outcomes’ definition can be found on their website (https://www.floridahealth.gov/statistics-and-data/survey-data/behavioral-risk-factor-surveillance-system/index.html).

### 2.2. GLMM Specification

Let *Y* be a binary health-related outcome (COPD as an example in the following description) from 2013 Florida BRFSS data. We constructed the following multilevel logistic regression model for a binary outcome, *Y*.

(1) $$P\left(Y_{ij}=1\right)={\text{logit}}^{-1}\left(X_{i}\beta +re_{j}\right)$$

In above formula,

*Y*_*ij*_: COPD that was answered as yes or no by respondent *i* from county *j* (*j* = 1, 2, …, 67).
*P*(*Y*_*ij*_ = 1) : the probability that the respondent has COPD.
*X*_*i*_ : *X* is a matrix of predictor variables, and *X*_*i*_ is the row of respondent *i*. There are three individual-level predictor variables: age (18 – 24 years, at 5-year intervals for 25 – 79 years, and 80 and above), sex (male and female), and race/ethnicity (Non-Hispanic white, black, American Indian or Alaska Native, Asian, Native Hawaiian/other Pacific Islander, other single race, and 2 or more races; and Hispanic) from the 2013 Florida BRFSS; and one county-level variable, percentage of the adult population below 150% of the poverty line from 2009–2013 American Community Survey (ACS, https://www.census.gov/programs-surveys/acs/data.html).
*β*: a fixed but unknown parameter vector.
*re*_*j*_ : the random effect for county *j*.

We used hierarchical Bayesian estimation and frequentist approaches, respectively, to estimate the parameters and simulate their distributions as below. All the analyses were implemented in SAS 9.4 (SAS Institute, Cary, NC).

### 2.3. Hierarchical Bayesian Estimation via MCMC

Model (1) was constructed in SAS by syntax of PROC MCMC, which utilizes MCMC methods to generate a large number of samples. We specified the prior distribution of *β* to be of the form ~*N*(0, 10,000), the prior distribution of *re*_*county*(*j*)_ as ~ *N*(0, $\sigma_{re}^{2}$), where $\sigma_{re}^{2}$ follows an inverse gamma distribution with shape 0.01 and scale 0.01. Good convergence for the model was achieved by simulating 100,000 iterations with another 10,000 “burn in”. To achieve independence of observations in the simulated posterior distributions, observations were thinned by a factor of 5. The model yielded the posterior distributions of parameters which contained *M* = 20,000 simulated values from all the iterations for each county.

For each iteration, parameter estimates ($\hat{\beta }$) and predicted random effects (${\hat{re}}_{j}$) from model (1) were applied to Florida county-level 2010 Decennial Census population counts. The population data were categorized by age (13 groups), sex (male and female), race/ethnicity (8 groups) for each county and was linked with ACS’ county-level percentage of the adult population below 150% of the poverty line, thus each county had a total of *m* population categories with a maximum 208 (13 × 2 × 8) (some counties lacked one or more categories). The predicted probability (${\hat{p}}_{kj}$) of developing COPD for the *k*^th^ population category in county *j* was calculated based on the following formula.

(2) $${\hat{p}}_{kj}=\text{exp}\left(X_{k}\hat{\beta }+{\hat{re}}_{j}\right)/\left(1+\text{exp}\left(X_{k}\hat{\beta }+{\hat{re}}_{j}\right)\right)$$

where

*X* is a matrix of demographic variables, and *X*_*k*_ is the row of population category *k*, and

$$X_{k}\hat{\beta }={\hat{\beta }}_{\text{age}}+{\hat{\beta }}_{\text{sex}}+{\hat{\beta }}_{\text{race}}+\text{poverty}*{\hat{\beta }}_{\text{poverty}}.$$

With ${\hat{p}}_{kj}$, we could calculate the estimated COPD through post-stratification for Florida state and for county *j* as below:

(3) $${\hat{P}}_{\text{state}}=\sum \left({\hat{p}}_{kj}*N_{j}\right)/\sum N_{j}\;{\hat{P}}_{\text{county}(j)}=\sum_{k=1}^{m}\left({\hat{p}}_{kj}*N_{kj}\right)/\sum_{k=1}^{m}N_{kj}$$

where *N*_*j*_ is the population in county *j*; *m* is the total population categories in county *j*, and *N*_*kj*_ is the population in the *k*^th^ category of county *j*.

By repeating (2) and (3), posterior distributions of ${\hat{P}}_{\text{state}}$ and ${\hat{P}}_{\text{county}(j)}$ for COPD were obtained. From the posterior distribution, the mean and 95% credible interval of ${\hat{P}}_{\text{state}}$ and ${\hat{P}}_{\text{county}(j)}$ were determined.

### 2.4. Frequentist Approaches

Model (1) was constructed with 2013 Florida BRFSS data and the residual subject-specific pseudo-likelihood method was used to produce $\hat{\beta }$ (with variance ${\hat{\sigma }}_{\hat{\beta }}$) and empirical best linear unbiased predictors, ${\hat{re}}_{j}$ (with variance ${\hat{\sigma }}_{{\hat{re}}_{j}}$). To simulate their distributions, we used the following methods, respectively.

#### 2.4.1. Monte Carlo Simulation

The idea behind this approach is to use a random number process to create repeated samples of $\hat{\beta }$ and ${\hat{re}}_{j}$ obtained from model (1) given their normal distributions. Thus Formula (2) was specified as:

(4) $${\hat{p}}_{kj}=\text{exp}\left(X_{k}{\hat{\beta }}^{*}+{\hat{re}}_{j}^{*}\right)/\left(1+\text{exp}\left(X_{k}{\hat{\beta }}^{*}+{\hat{re}}_{j}^{*}\right)\right)$$

where ${\hat{\beta }}^{*}$ is a normal variate with mean $\hat{\beta }$ and variance ${\hat{\sigma }}_{\hat{\beta }}$ and ${\hat{re}}_{j}^{*}$ is a normal variate with mean ${\hat{re}}_{j}$ and variance ${\hat{\sigma }}_{{\hat{re}}_{j}}$. Model (4) was repeated for 1000 times and then applied each ${\hat{p}}_{kj}$ to Formula (3) which generated the 1000 ${\hat{P}}_{\text{county}(j)}$. The mean and 95% CIs (a range between the 2.5^th^ and 97.5^th^ values) were determined.

#### 2.4.2. Parametric Bootstrapping

The difference of this approach with non-parametric bootstrapping is the source of the bootstrap samples. In non-parametric bootstrapping, the bootstrap samples were drawn from the original 2013 Florida BRFSS data; while in parametric bootstrapping, bootstrap data were drawn from the model fitted to 2013 Florida BRFSS data. We adopted Zhang *et al*.’s [13] revised algorithm as follows.

Step 1. Model (1) was built using Florida BRFSS data from which predicted linear predictor (${\hat{\eta }}_{ij}=X_{i}\hat{\beta }+{\hat{re}}_{j}$) and the standard error (${\hat{\sigma }}_{ij}$) of ${\hat{\eta }}_{ij}$ were obtained.
Step 2. A random sample of ${\hat{\eta }}_{ij}^{*}$ was taken from a normal distribution with mean ${\hat{\eta }}_{ij}$ and variance ${\hat{\sigma }}_{ij}^{2}$; and the predicted probability (${\hat{p}}_{ij}^{*}=\frac{e^{{\hat{\eta }}_{ij}^{*}}}{1+e^{{\hat{\eta }}_{ij}^{*}}}$) was calculated. With ${\hat{p}}_{ij}^{*}$, we could generate a bootstrap sample *B* of COPD (${\hat{y}}_{ij}^{*}=1$ or 0) for each 2013 FLORIDA BRFSS respondent and use it to refit model (1). Predicted probability ${\hat{p}}_{kj}$ was obtained through the model for this bootstrap sample and was used to generate ${\hat{P}}_{\text{state}}$ and ${\hat{P}}_{\text{county}(j)}$ of COPD using Formula (3). Step 2 was repeated for 1000 times to compute the mean and 95% CI by taking the 2.5^th^ and 97.5^th^ values for ${\hat{P}}_{\text{state}}$ and ${\hat{P}}_{\text{county}(j)}$ of COPD.

#### 2.4.3. Non-Parametric Bootstrapping

Non-parametric bootstrapping is a resampling technique to estimate statistics (means, medians, SEs, and percentiles) by sampling the original dataset with replacement. The bootstrap sample usually has the same size as the original dataset. Specific steps of non-parametric bootstrapping in this study follow:

Step 1. PROC SURVEYSELECT in SAS was used to resample2013 Florida BRFSS data (*B* = 1000 times) randomly with replacement to form *B* bootstrap samples. In this procedure, we stratified random samples by county to ensure each county had the sample size as the original Florida BRFSS dataset.
Step 2. We put all *B* random bootstrap samples into a single data set. For each bootstrap sample dataset, model (1) was constructed to obtain predicted probability ${\hat{p}}_{kj}$. Thus we could obtain ${\hat{P}}_{\text{county}(j)}$ and ${\hat{P}}_{\text{state}}$ of COPD using Formulas (3) for this sample. The mean of ${\hat{P}}_{\text{county}(j)}$ for COPD was calculated over 1000 samples and 95% CI was taken as a range between the 2.5^th^ and 97.5^th^ values.

The performance of frequentist approaches was evaluated based on how their estimates and 95% CIs were close to estimates and 95% credible intervals generated from Bayesian estimation as well as how much their computation time was consumed.

## 3. Results

### 3.1. State-Level Estimates and Statistical Intervals Comparison

The mean estimates and 95% statistical intervals for each of the health-related outcomes at the state level are presented in Table 1. It shows that the mean estimates at the state level in Florida were similar across all approaches for each outcome. The 95% CIs differed substantially, though. For estimated COPD, the 95% CIs generated by MC simulation (7.1% – 7.8%), parametric bootstrapping (7.1% – 7.8%), and non-parametric bootstrapping (6.8% – 7.5%) were all close to the credible intervals generated by Bayesian estimation via MCMC (6.9% – 7.6%); the 95% CIs of the direct survey estimate were comparatively wider (6.9% – 7.9%). Similar patterns were observed for binge drinking and arthritis as well.

### 3.2. County-Level Estimates and Statistical Intervals Comparison

**Figure 1.**
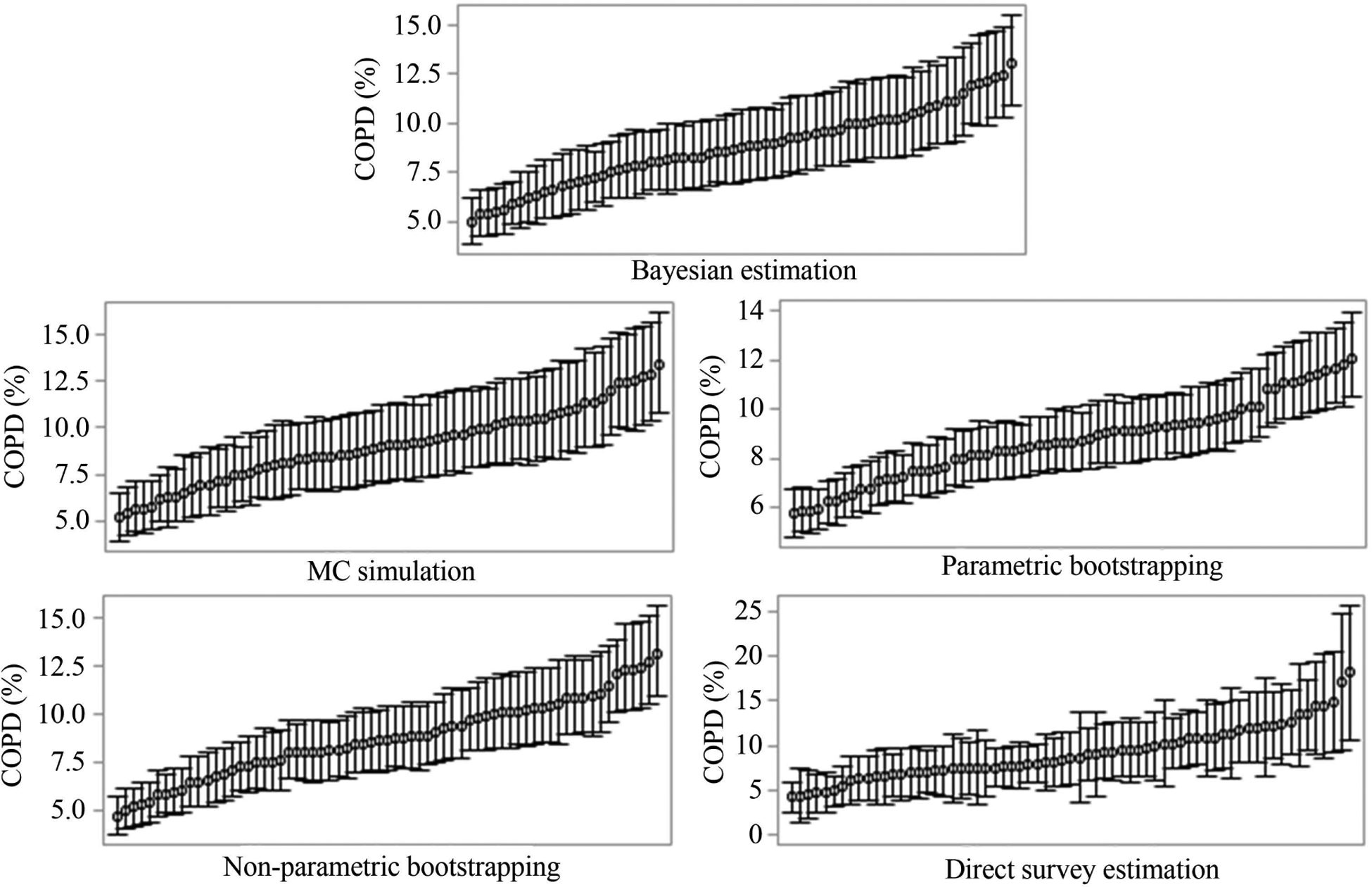
The mean estimates and 95% credible incidence (Bayesian estimation) and 95% CIs (other approaches) for COPD in 67 counties, Florida.

**Figure 2.**
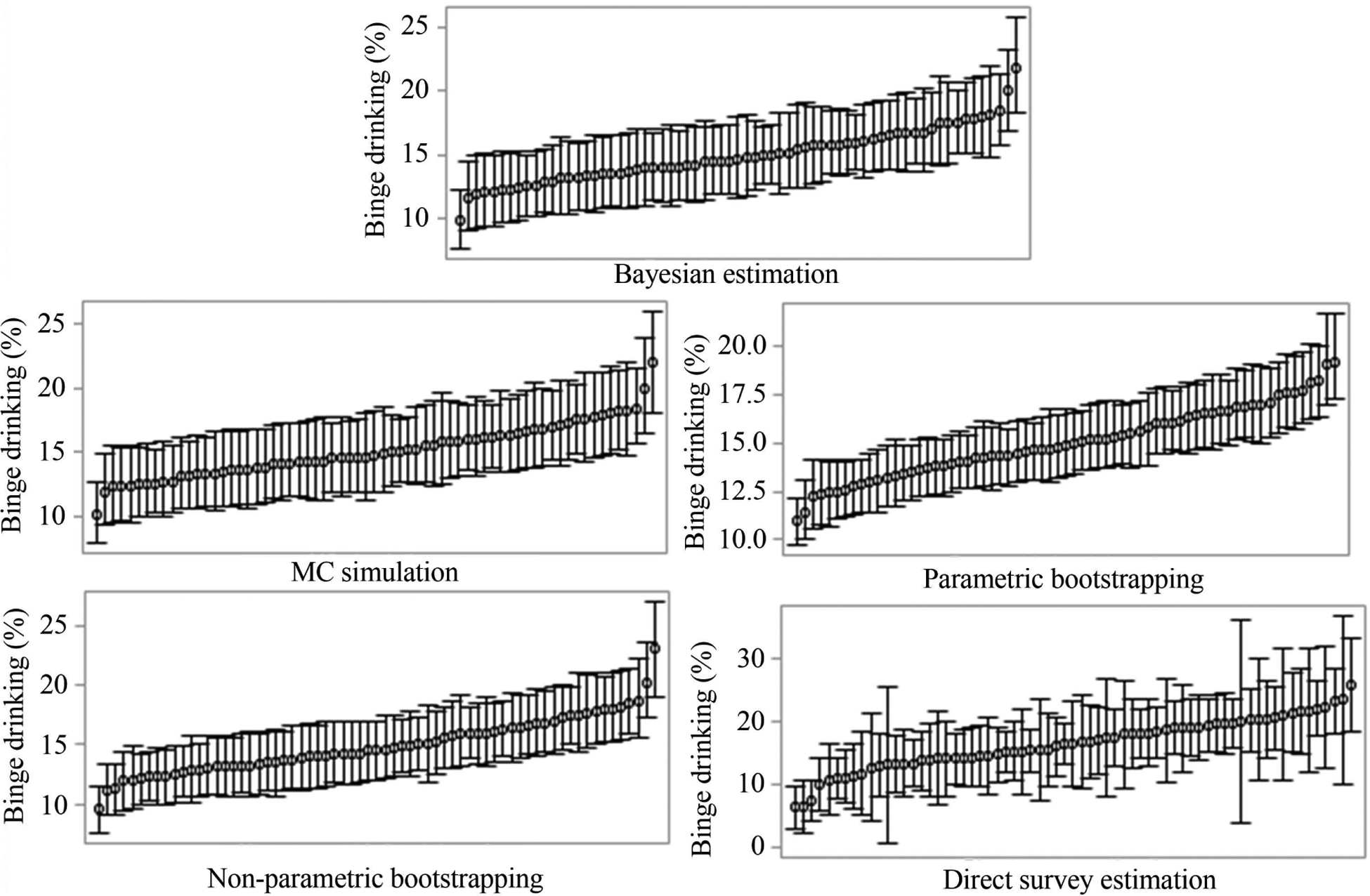
The mean estimates and 95% credible incidence (Bayesian estimation) and 95% CIs (other approaches) for binge drinking in 67 counties, Florida.

**Figure 3.**
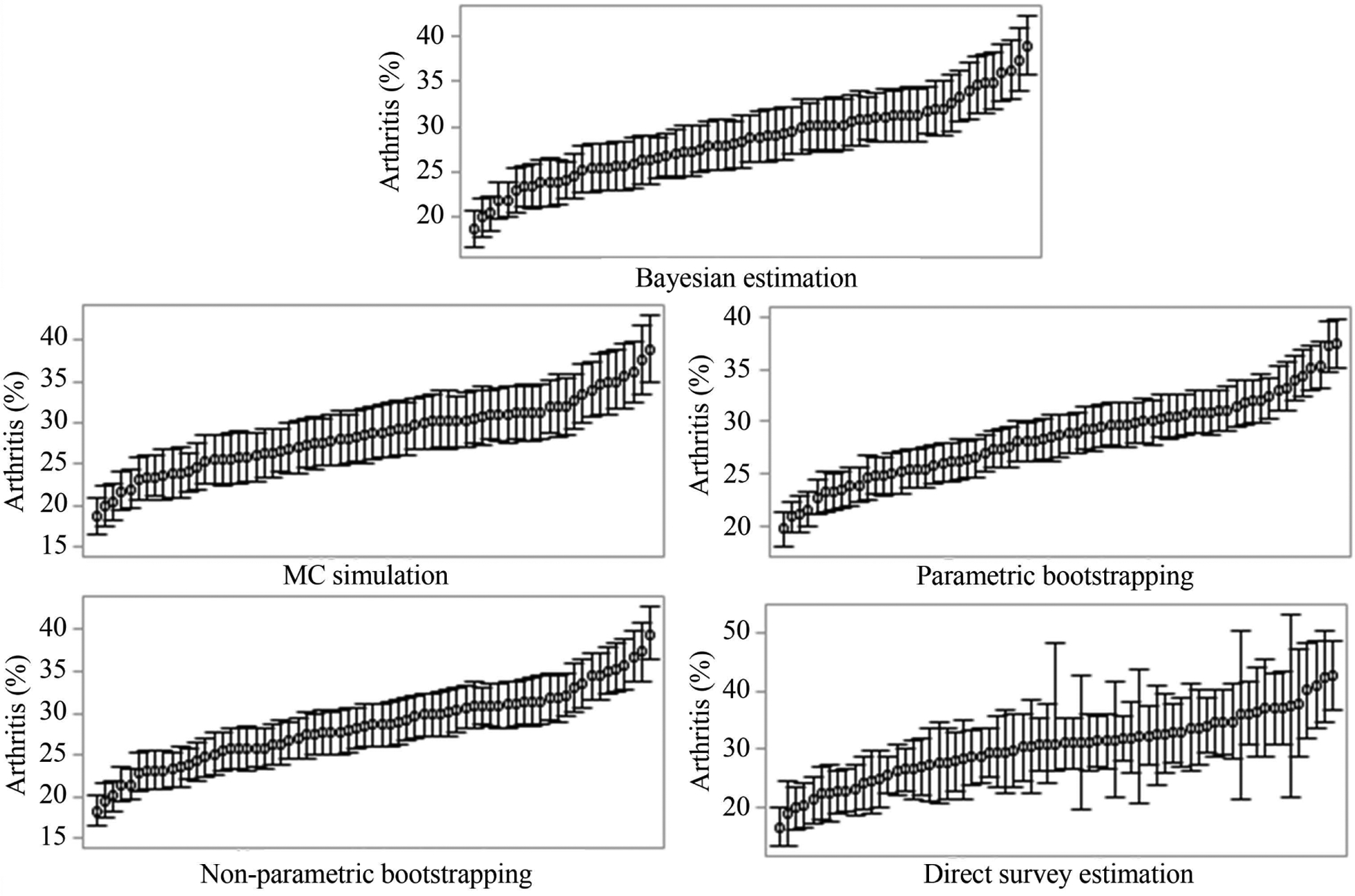
The mean estimates and 95% credible incidence (Bayesian estimation) and 95% CIs (other approaches) for arthritis in 67 counties, Florida.

Figure 1 illustrates the ranked mean estimates and 95% statistical intervals of COPD for all the 67 counties. It shows that MC simulation and non-parametric bootstrapping produced similar 95% CIs with 95% credible incidence produced by Bayesian estimation for all the county-level estimates. In comparison, parametric bootstrapping yielded much narrower CIs; while the direct survey estimation generated much wider CIs for some counties. The distributions of estimates for binge drinking (Figure 2) and arthritis (Figure 3) showed a similar pattern to COPD (Figure 1). The total computational time taken by different approaches was shown in Table 2. The time was the total time for all the analysis of MRP, including the model construction and post-stratification, for both state- and county-level estimation. As MC simulation approach did not need to refit models, it was more computationally efficient.

## 4. Discussion

In this study, we generated point estimates for each of the selected outcomes and simulated their distributions via an SAE application in a health survey. As expected, we observed similar mean estimates for each outcome but different intervals across all the approaches at both state and county levels. The method of MC simulation and non-parametric bootstrapping yielded the closest 95% CIs to credible intervals by Bayesian estimation for all the selected outcomes, but MC simulation was much more computationally efficient than the others.

To generate a proper statistical interval, an approach needs to account for three sources of uncertainty: the residual variance, the uncertainty in the fixed effects parameter estimation, and the uncertainty in the variance parameters for the random effects. In full Bayesian analysis, one uses probability distributions (prior distribution and data likelihood) to model the credibility of possible parameter values. The outcome of Bayesian analysis, the posterior, models the probability of each possible parameter value being true given the prior and likelihood [14], so it takes fuller account of uncertainties and therefore it yields reasonable credible intervals when applied to SAE. However, in public health practice, the frequentist approach is often used and may continue to be dominant because the common criticism of Bayesian method is its specification of prior distribution and computational intensity [2].

In the framework of model-based estimation, parametric bootstraps for linear mixed models have been introduced to estimate mean squared error (MSE) by Laird and Louis [15], Pfeffermann and Tiller [16], Butar and Lahiri [17]; non-parametric bootstraps were proposed by Pfeffermann and Tiller [16]; double bootstraps were also proposed by several researchers [18] [19] [20]. Bootstraps were also used to estimate MSE in logistic mixed model for small area estimation [21]. The essence of parametric bootstrapping is the drawing of pseudo-samples from a model fitted to the original sample [22]. Although it incorporates the variability of the model parameters, it only uses a finite sample of the original data to approximate the estimator’s distribution [9]. Therefore, it yielded comparatively narrower CIs which would not change substantially by increasing the bootstrap simulation B but could be related to the prevalence of the outcome. Non-parametric bootstrapping achieved similar CIs around the mean as Bayesian estimation. Novkaniza *et al*. found that parametric bootstrapping’s CIs was narrower than nonparametric bootstrapping’s CIs for small area estimates as well [10]. Both bootstrapping, however, were also computationally intensive and is not efficient for large scale small area estimation in a public health application.

For example, the U.S. CDC’s PLACES Project (www.cdc.gov/PLACES) provided model-based estimates for 27 measures of chronic disease, health-related behavior, and prevention service at U.S. county, incorporated place, ZIP Code Tabulation Area, and census tract levels using the nationwide BRFSS, as opposed to just a single state’s BRFSS as the data source in this study. Given the large dataset with more than 400,000 respondents per year and complex data structure of the nationwide BRFSS, inclusion of multiple measures, along with multiple geographic area levels, the full Bayesian hierarchical model would be incredibly high-dimensional and extremely time consuming; the model construction could run out of the computational memory and the entire process would be very impractical to implement. MC simulation turned out to be a more reasonable and practical choice because it not only produced similar CIs to the credible intervals generated from Bayesian hierarchical models, but it also was more efficient in terms of computational time.

This study is subject to several limitations. It should be noted that the mechanisms of Bayesian and frequent approaches are inherently different. Therefore, the intervals generated by hierarchical Bayesian models simply play a role of “benchmark value” for comparison purpose. Second, this study only focused on the sampling approaches, but there are some other techniques to create statistical intervals. Third, as we run into an “out of computational memory” issue when we attempted to use nationwide BRFSS as an illustration, we had to select a subset of BRFSS.

## 5. Conclusion

Statistical intervals may help local health departments identify if different counties have different sources and needs, or if different sub-population groups in the small areas are equally exposed to a particular disease. Different approaches may produce different intervals as shown in this study. Given their comparison with the Bayesian estimation and their computational performance, the MC simulation approach produced reasonable CIs for multilevel model-based small area estimates but was much simpler to implement and could be applied as a suitable option for public health practice.

## Figures and Tables

**Table 1.** The means and 95% statistical intervals of the state-level estimates (%) for each outcome by different approaches for Florida, 2013.

	COPD		Binge drinking		Arthritis	
	Mean	95% interval^1^	Mean	95% interval^1^	Mean	95% interval^1^
Bayesian estimation, model-based	7.2	6.9, 7.6	15.5	14.9, 16.2	25.7	25.1, 26.3
MC simulation, model-based	7.5	7.1, 7.8	15.7	15.1, 16.4	25.8	25.2, 26.4
Parametric bootstrapping, model-based	7.4	7.1, 7.8	15.6	14.8, 16.0	26.0	25.5, 26.6
Non-parametric bootstrapping, model-based	7.4	6.8, 7.5	15.3	15.0, 16.3	25.5	24.9, 26.1
Direct survey estimate	7.4	6.9, 7.9	15.6	14.6, 16.6	26.0	25.1, 26.9

1. 95% intervals are credible incidence (Bayesian estimation) or confidence intervals (other approaches).

**Table 2.** Total computational time (seconds) of different approaches for each of the outcomes.

	Model construction	Post-stratification
Bayesian estimation	10,800	23,400
MC simulation	12	960
Parametric bootstrapping	1800	42
Non-parametric bootstrapping	1800	42
